# Core signaling pathways in ovarian cancer stem cell revealed by integrative analysis of multi-marker genomics data

**DOI:** 10.1371/journal.pone.0196351

**Published:** 2018-05-03

**Authors:** Tianyu Zhang, Jielin Xu, Siyuan Deng, Fengqi Zhou, Jin Li, Liwei Zhang, Lang Li, Qi-En Wang, Fuhai Li

**Affiliations:** 1 Department of BioMedical Informatics (BMI), The Ohio State University, Columbus, Ohio, United States of America; 2 School of Mathematical Sciences, Dalian University of Technology, Dalian, China; 3 Department of Radiology, The Ohio State University, Columbus, Ohio, United States of America; Università degli Studi della Campania, ITALY

## Abstract

Tumor recurrence occurs in more than 70% of ovarian cancer patients, and the majority eventually becomes refractory to treatments. Ovarian Cancer Stem Cells (OCSCs) are believed to be responsible for the tumor relapse and drug resistance. Therefore, eliminating ovarian CSCs is important to improve the prognosis of ovarian cancer patients. However, there is a lack of effective drugs to eliminate OCSCs because the core signaling pathways regulating OCSCs remain unclear. Also it is often hard for biologists to identify a few testable targets and infer driver signaling pathways regulating CSCs from a large number of differentially expression genes in an unbiased manner. In this study, we propose a straightforward and integrative analysis to identify potential core signaling pathways of OCSCs by integrating transcriptome data of OCSCs isolated based on two distinctive markers, ALDH and side population, with regulatory network (Transcription Factor (TF) and Target Interactome) and signaling pathways. We first identify the common activated TFs in two OCSC populations integrating the gene expression and TF-target Interactome; and then uncover up-stream signaling cascades regulating the activated TFs. In specific, 22 activated TFs are identified. Through literature search validation, 15 of them have been reported in association with cancer stem cells. Additionally, 10 TFs are found in the KEGG signaling pathways, and their up-stream signaling cascades are extracted, which also provide potential treatment targets. Moreover, 40 FDA approved drugs are identified to target on the up-stream signaling cascades, and 15 of them have been reported in literatures in cancer stem cell treatment. In conclusion, the proposed approach can uncover the activated up-stream signaling, activated TFs and up-regulated target genes that constitute the potential core signaling pathways of ovarian CSC. Also drugs and drug combinations targeting on the core signaling pathways might be able to eliminate OCSCs. The proposed approach can also be applied for identifying potential activated signaling pathways of other types of cancers.

## Introduction

Over 90% of ovarian cancers are epithelial in origin, and epithelial ovarian cancer, especially the most aggressive subtype high-grade serous ovarian cancer (HGSOC), accounts for the majority of ovarian cancer deaths [1, 2]. Most tumors are initially responsive to the conventional chemotherapy, and the patients enter into clinical remission after initial treatment. However, tumor recurrence occurs in more than 70% of patients despite treatment, and the majority eventually becomes refractory to treatments [3]. Recent research evidences show that tumor is a mixture of heterogeneous populations of cells with different levels of malignity. A subpopulation of tumor cells characterized by the capacity of self‐renewing, differentiation, and tumor‐initiating are called cancer stem cells (CSCs) or tumor initiating cells (TICs) [4]. CSCs play important roles in tumor initiation, progression, metastasis, recurrence and drug resistance [4–7]. Thus, elimination of CSCs is important to overcome drug resistance to improve the prognosis of cancer patients. The knowledge about CSCs is limited, and one major challenge is that it is difficult to identify and isolate CSCs with few biomarkers because CSCs are heterogeneous and could exist a specific hierarchy [8–10]. Ovarian cancer stem cells (OCSCs) have been successfully identified and isolated based on their expression of distinctive cell surface markers CD44, CD117, MyD88, and CD133 [11, 12], as well as the activity of ALDH [13]. These CSCs harbor enhanced tumorigenicity and chemoresistance [14]) and are thought to drive the universal recurrence of ovarian cancer, as well as responsible for the development of therapeutic resistance [15]. Though studies with these markers show evidence in support of OCSCs [16], there is still a lack of effective drugs to differentiate and eliminate them [17].

Though common signaling pathways, e.g., WNT, NOTCH, SHH, JAK/STAT, have been associated with all types of CSCs [18, 19], the core signaling mechanism regulating Ovarian CSCs remain unclear. The differential gene expression analysis often fails to identify genes regulate CSCs because it is difficult to identify a few testable targets from a large number of differentially expressed genes mixed with many passenger genes. Additionally, important proteins regulating CSCs might be missed because either the fold change is small or the gene expression data is not available. Therefore, it is necessary to integrate multi-datasets with prior knowledge, e.g., regulatory network and signaling pathways, to increase the possibility of identifying the true CSC driver genes in the systems biology perspective. Thus, in this study, we propose an approach to identify potential core signaling pathways of OCSCs by integrating transcriptome data of OCSCs isolated based on two distinctive markers, ALDH and side population (Hoechst 33342 stain), with the prior knowledge of regulatory network and KEGG signaling pathways. Our hypothesis is that the integrative analysis of multi-genomics data sets of OCSCs with distinct markers could infer more accurate driver-signaling network regulating CSC to generate a small number of testable biomarkers and drugs. In specific, we first identify the common activated transcription factors (TFs) in two OCSC populations; and then construct the core signaling pathways by uncovering the up-stream signaling cascades of the activated TFs, which constitute the potential core signaling pathways of ovarian CSC. Drugs targeting on the up-stream signaling cascades of activated TFs are selected as potential treatments to eliminate ovarian cancer stem cells. The details of methodology and datasets are introduced in Section 2; and analysis results are shown in Section 3, followed by the discussions and conclusion.

## Materials and methodology

### Transcriptome datasets of OCSCs

In this study, we manually searched the ovarian CSC gene expression datasets in NCBI GEO (Gene Expression Omnibus) using the aforementioned markers and “Ovarian” as the keywords, e.g., “CD44, Ovarian”, and only two datasets were found, i.e., GSE33874 and GSE82304. The dataset GSE33874 is the gene expression profile of isolated side population (SP-Hoechst Blue High and Hoechst Red Low, Ovarian CSCs) and main population (MP) of fresh ascites obtained from women with high-grade advanced stage papillary serous ovarian adenocarcinoma [20]. Gene expression profiles (in triplicate) of SKOV3 human ovarian cancer cells of Aldefluor high (ALDH+, Ovarian CSCs) and Aldefluor low (ALDH-) populations were available in dataset GSE82304 [16]. The GEO2R was employed to get the fold change data of individual genes.

### KEGG signaling pathways and regulatory network

To obtain the KEGG signaling pathways, the “Pathview” R package was employed to download KGMLs of humans pathways [21]. Then the “KEGGgraph” R package was used to extract nodes and edges of KEGG signaling pathways from KGMLs [22]. In total, 282 signaling pathways were collected from seven categories: metabolism, genetic information processing, environmental information processing, cellular processes, organismal systems, human diseases, and drug development. The TF-Target regulatory network was downloaded from the supplemental material of reference [23], which was derived from the TF binding site predictions for all target genes from TRANSFAC (v7.4) [24]. In summary, the TF-target regulatory network consists of 230 TFs, 12733 target genes, and 79100 TF-Target interactions.

### Signaling pathway construction

First, the Fisher’s exact test (using hypo-geometric distribution) [25] was used to identify the activated TFs by comparing the number of up-regulated targets vs. the number of all target genes, with the number of all the up-regulated genes vs. the number of all the genes tested. The p-value threshold, 0.05, was used to select the activated TFs. Second, all 282 signaling pathways from KEGG were examined, and all the signaling cascades from the starting nodes to the activated TFs were extracted, and then the top 3 signaling paths were kept to construct the signaling pathway regulating the given TFs. The python package, NetworkX, was used to screen all the 282 KEGG signaling pathways to extract signaling cascades starting from the beginning genes of individual signaling pathways to the given TFs. Then we score each signaling cascades using the average expression fold change of genes (on the signaling cascades and with fold change > 0). To control the size of up-stream signaling network of given TFs, the top 3 signaling cascades are kept. The up-regulated target genes (Fold change > = 2) in both datasets are linked to the given TFs.

## Results

### Twenty-two activated TFs

In the two gene expression datasets, there are 1988 and 2528 up-regulated genes (fold change > = 2); and 883 and 2821 down-regulated genes (fold change < = 0.5) respectively. It is difficult for biologists to identify potential targets associated with Ovarian CSCs from such large number of differentially expressed genes. With the aim of discovering testable regulatory signaling networks that maintain Ovarian CSCs, we identify the activated TFs (whose target genes are up-regulated) in both datasets (CSCs isolated from ALDH^+^ marker and side-population) using the Fisher’s exact test by integrating the up-regulated genes (Fold_Change > = 2) and the TF-target interactome (gene regulation network) data. In total, 22 TFs are identified (see **Table 1**). As can be seen, some TFs will be missed using only gene expression fold change because either there is no gene expression data available (NA) or the fold change is small. We conducted the literature search to evaluate these TFs, and surprisingly 15 TFs have been reported to play important roles in cancer stem cell regulation (see **Table 2**). For example, FOXO3 is essential for maintenance of CSC properties in pancreatic ductal adenocarcinoma [26]; FOXO4 is related to stem cell-like properties of large B-cell lymphoma cells [27]. LEF1 is able to regulate glioblastoma stem-like cell self-renewal [28]; NFATc2 enhances tumor-initiating phenotypes in lung adenocarcinoma [29].

**Table 1:** Twenty-two activated TFs (with *p*_value < = 0.05 in Fisher’s exact test). The *p*_value is obtained from Fisher’s exact test in dataset_1 and dataset_2; and Log_FC denotes the log scaled gene expression fold change (CSC vs. non-CSC) in two datasets; and Within_KEGG indicates if the given transcription factor is on some signaling cascades from KEGG signaling pathways.

TranscriptionFactors	*p*_value (dataset_1)	*p*_value (dataset_2)	Log_FC (dataset_1)	Log_FC (dataset_2)	Within_KEGG
**ELK1**	2.05E-07	8.90E-07	0.48	0.12	Y
**FOXA1**	0.00012	0.00034	1.03	2.63	
**NRF1**	0.01100	0.00047	1.59	2.49	Y
**NR3C1**	0.01945	0.00076	0.51	-2.16	
**FOXL1**	0.00295	0.00160	0.30	0.79	
**FOXO4**	0.00243	0.00191	0.53	0.75	Y
**TAL1_TCF4**	0.01586	0.00285	NA	NA	
**LEF1**	0.00186	0.00424	-1.05	-1.02	Y
**GABPB1**	0.00062	0.00761	0.14	-0.43	
**MEF2A**	0.02146	0.00967	-0.73	0.82	Y
**FOXI1**	0.00219	0.01115	0.37	-3.00	
**FOXJ2**	0.00032	0.01361	-0.60	-0.49	
**FOXO3**	0.00094	0.01948	-0.92	0.73	Y
**FOXJ1**	0.00057	0.02170	0.10	NA	
**POU2F1**	0.00037	0.02244	-1.55	-0.32	
**NFATC2**	0.00405	0.02352	-1.42	NA	Y
**SPI1**	0.03029	0.02423	0.80	-0.02	Y
**POU3F2**	0.00639	0.03073	-0.44	-1.85	
**S8**	1.80E-06	0.04148	NA	NA	
**TEAD1**	0.00230	0.04209	-0.59	-0.04	Y
**FOX**	0.00028	0.04483	NA	NA	
**E2F1**	0.02243	0.04751	0.37	0.22	Y

**Table 2:** Literature reports of the Twenty-two TFs. Fifteen TFs have been reported to play important roles in cancer stem cells.

Transcription Factors	Titles of articles related to CSC
**ELK1**	MZF-1/Elk-1 interaction domain as therapeutic target for protein kinase Cα-based triple-negative breast cancer cells. [30]
**FOXA1**	FOXA1 expression affects the proliferation activity of luminal breast cancer stem cell populations. [31]
**NRF1**	Transcriptional regulation of chemokine receptor 4 (CXCR4) by nuclear respiratory factor 1 (NRF1) controls estrogen-induced malignant transformation of breast epithelial cells to breast cancer stem cells. [32]
**NR3C1**	Haploinsufficiency for NR3C1, the gene encoding the glucocorticoid receptor, in blastic plasmacytoid dendritic cell neoplasms. [33]
**FOXL1**	Wnt/b-catenin signaling in cancer stemness and malignant behavior. [34]
**FOXO4**	FOXO4 expression is related to stem cell-like properties and resistance to treatment in diffuse large B-cell lymphoma. [27]
**TAL1_TCF4**	A Small-Molecule Antagonist of the β-Catenin/TCF4 Interaction Blocks the Self-Renewal of Cancer Stem Cells and Suppresses Tumorigenesis. [35]
**LEF1**	LEF1 regulates glioblastoma cell proliferation, migration, invasion, and cancer stem-like cell self-renewal. [28]
**GABPB1**	Resetting cancer stem cell regulatory nodes upon MYC inhibition. [36]
**MEF2A**	
**FOXI1**	
**FOXJ2**	
**FOXO3**	FOXO3/PGC-1β signaling axis is essential for cancer stem cell properties of pancreatic ductal adenocarcinoma. [26]
**FOXJ1**	
**POU2F1**	
**NFATC2**	Cancer-stem-cell (CSC) marker, DCLK1-S, enhances invasive potential of cancer cells by phosphorylating/activating NFATc2: role of COL3A1 and SPARC in mediating metastatic effects of DCLK1-S/NFATc2. [29]
**SPI1**	Inhibition of the transcription factor Sp1 suppresses colon cancer stem cell growth and induces apoptosis in vitro and in nude mouse xenografts. [37]
**POU3F2**	
**S8**	
**TEAD1**	YAP/TEAD Co-Activator Regulated Pluripotency and Chemoresistance in Ovarian Cancer Initiated Cells. [38]
**FOX**	Identification of chromatin accessibility domains in human breast cancer stem cells. [39]
**E2F1**	Transcriptional control of stem cell fate by E2Fs and pocket proteins. [40]

### Up-stream signaling cascades regulating activated TFs

**Fig 1:**
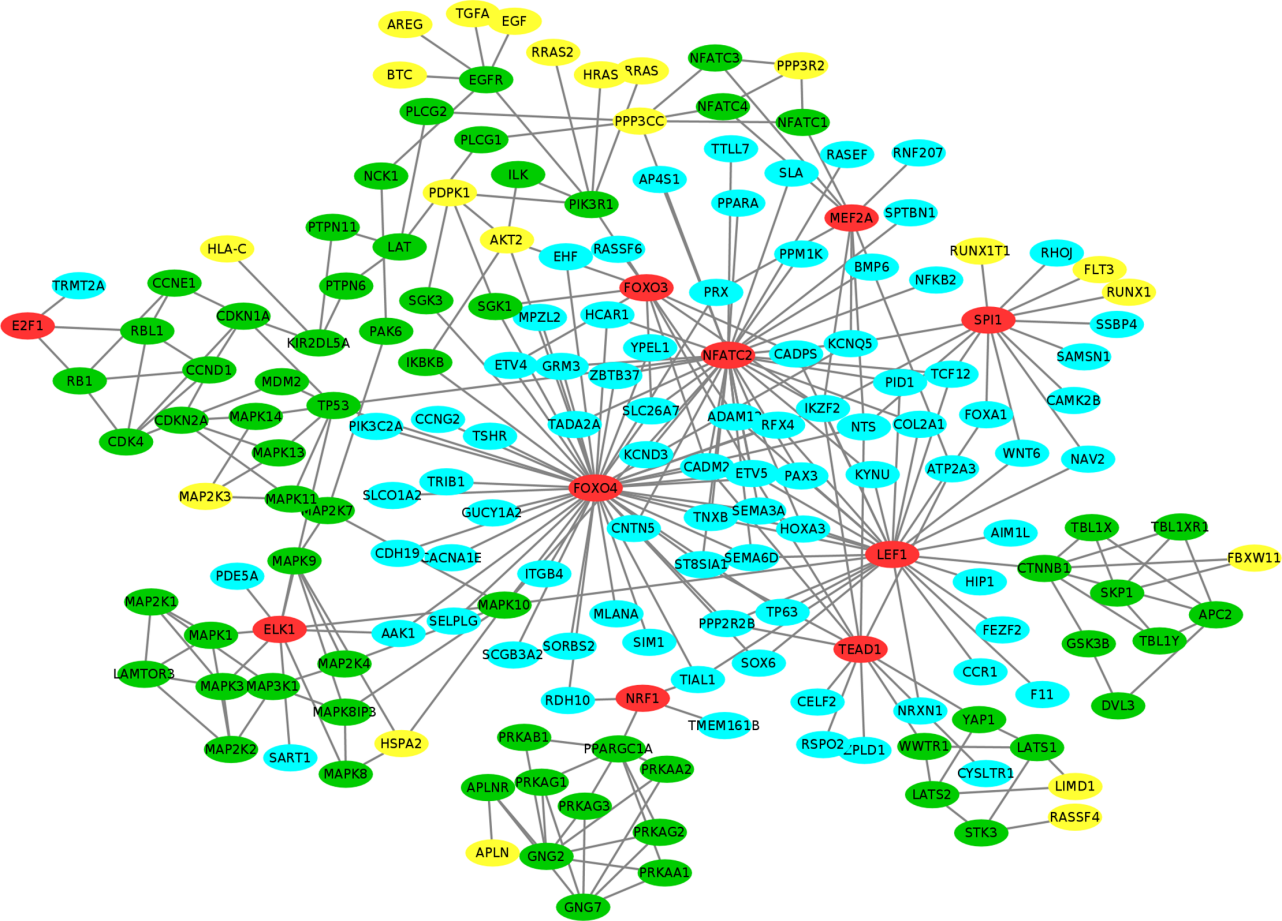
Activated signaling pathways of ovarian CSCs. The color of yellow, green, red and cyan represents signaling starting genes, signaling transduction genes, TFs and target genes respectively.

We further uncover the up-stream signaling cascades regulating these activated TFs using KEGG signaling pathways. Out of 22 activated TFs, 10 TFs (ELK1, NRF1, FOXO4, LEF1, MEF2A, FOXO3, NFATC2, SPI1, TEAD1 and E2F1) are found in KEGG signaling pathways with up-stream signaling cascades link to them (see **Table 1** and red color nodes in **Fig 1**). As can be seen in **Fig 1**, many target genes (cyan color nodes) of transcription factors, FOXO4, LEF1, NFATC2, SPI1 and TEAD1, are up-regulated (fold_change > = 2) in both datasets. The yellow color nodes represent the starting proteins activating the signaling cascades in KEGG, e.g., the MAP kinases that are often activated by mitogenic and environmental stress, the EGF growth factor, and FLT3 that encodes a class III receptor tyrosine kinase that regulates hematopoiesis, and PPP3R2 that is related to the calcium signaling. These signaling cascades provide potential testable biomarkers of Ovarian CSCs for experimental design.

### FDA approved drugs targeting on up-stream signaling of activated TFs

**Fig 2:**
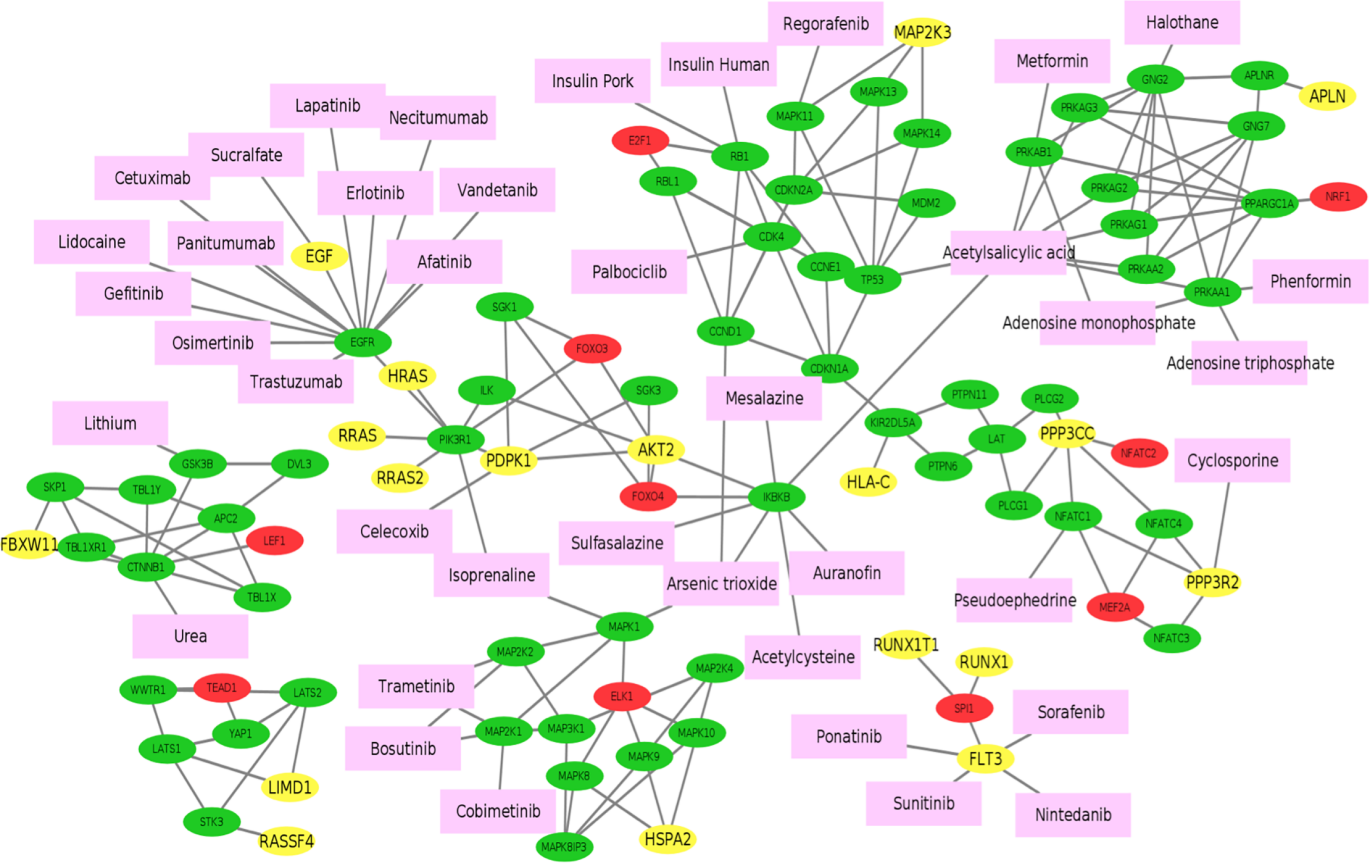
FDA approved drugs targeting on up-stream signaling of activated TFs of ovarian CSCs. The color of yellow, green, red, cyan and pink represents signaling starting genes, signaling transduction genes, TFs, target genes, drugs respectively.

To investigate potential drugs that can perturb the Ovarian CSCs, we mapped the FDA approved drugs on the integrative signaling network (see **Fig 2**). The target information obtained from DrugBank (version 5.0.11) [41]. In total, 40 drugs (pink nodes in **Fig 2**) were selected targeting on different signaling cascades. Through the literature search, we found that 15 drugs have been reported to treat cancer stem cells (see **Table 3**). For example, Palbociclib can block the propagation of lung, ovarian and breast cancer stem cells by targeting on CDK4 [42]. Bosutinib and Trametinib targeting on the MAP2K1/2 were used for cancer stem cell and multi-drug resistance treatment [43, 44]. Celecoxib was used in colon cancer stem cell related treatment by targeting on PDPK1 [45]. Metformin and Phenformin (targeting on PRKAA1/PRKAB1 were used for eliminate prostate cancer stem cells [46, 47]. Moreover, Metformin has been shown to be able to overcome drug resistance to tyrosine kinase inhibitors (TKI) of EGF receptor (EGFR) in lung cancer [48]. **Table 3** shows more details. In addition to the single drug treatment, combinations of drugs targeting on the different signaling cascades might have better effects to eliminate cancer stem cells. In summary, this analysis can provide testable hypothesis and potential drug candidates to eliminate OCSCs.

**Table 3:** FDA approved drugs targeting on upstream signaling of TFs.

Drug Name	Target	Titles of articles related to CSC treatment
**Palbociclib**	CDK4	Targeting cancer stem cell propagation with palbociclib, a CDK4/6 inhibitor: Telomerase drives tumor cell heterogeneity. [42]
**Arsenic trioxide**	CCND1 MAPK1 IKBKB	Arsenic trioxide sensitizes cancer stem cells to chemoradiotherapy. A new approach in the treatment of inoperable glioblastoma multiforme. [49]
**Insulin Human**	RB1	
**Insulin Pork**	RB1	
**Regorafenib**	MAPK11	
**Acetylsalicylic acid**	TP53 IKBKB PRKAG2 PRKAG3 PRKAA1 PRKAA2 PRKAG1 PRKAB1	
**Cobimetinib**	MAP2K1	
**Bosutinib**	MAP2K1 MAP2K2	The therapeutic potential of targeting ABC transporters to combat multi-drug resistance. [43]
**Trametinib**	MAP2K1 MAP2K2	Roles of EGFR and KRAS and their downstream signaling pathways in pancreatic cancer and pancreatic cancer stem cells. [44]
**Isoprenaline**	MAPK1PIK3R1	
**Celecoxib**	PDPK1	Expression Patterns of Cancer Stem Cell Markers During Specific Celecoxib Therapy in Multistep Rat Colon Carcinogenesis Bioassays. [45]
**Sucralfate**	EGF	
**Cetuximab**	EGFR	Antitumor activity of Cetuximab in combination with Ixabepilone on triple negative breast cancer stem cells. [50]
**Trastuzumab**	EGFR	Cancer stem cell-driven efficacy of trastuzumab (Herceptin): towards a reclassification of clinically HER2-positive breast carcinomas. [51]
**Lidocaine**	EGFR	
**Gefitinib**	EGFR	
**Erlotinib**	EGFR	Tyr1068-phosphorylated epidermal growth factor receptor (EGFR) predicts cancer stem cell targeting by erlotinib in preclinical models of wild-type EGFR lung cancer. [52]
**Lapatinib**	EGFR	
**Panitumumab**	EGFR	Cancer Stem Cell-Based Models of Colorectal Cancer Reveal Molecular Determinants of Therapy Resistance. [53]
**Vandetanib**	EGFR	
**Afatinib**	EGFR	Afatinib radiosensitizes head and neck squamous cell carcinoma cells by targeting cancer stem cells. [54]
**Osimertinib**	EGFR	
**Necitumumab**	EGFR	
**Mesalazine**	IKBKB	Mesalazine inhibits the β-catenin signalling pathway acting through the upregulation of μ-protocadherin gene in colo-rectal cancer cells. [55]
**Sulfasalazine**	IKBKB	
**Auranofin**	IKBKB	
**Acetylcysteine**	IKBKB	
**Urea**	CTNNB1	
**Lithium**	GSK3B	
**Cyclosporine**	PPP3R2	Cancer Stem Cells in Prostate Cancer: Implications for Targeted Therapy. [56]
**Pseudoephedrine**	NFATC1	
**Halothane**	GNG2	
**Adenosine monophosphate**	PRKAA1 PRKAB1	
**Adenosine triphosphate**	PRKAA1	Extracellular ATP reduces tumor sphere growth and cancer stem cell population in glioblastoma cells. [57]
**Phenformin**	PRKAA1	Metformin and phenformin deplete tricarboxylic acid cycle and glycolytic intermediates during cell transformation and NTPs in cancer stem cells. [46]
**Metformin**	PRKAB1	Metformin and prostate cancer stem cells: a novel therapeutic target. [47]
**Sorafenib**	FLT3	
**Sunitinib**	FLT3	
**Ponatinib**	FLT3	
**Nintedanib**	FLT3	

## Discussion and conclusion

Most ovarian cancer tumors are initially responsive to the conventional chemotherapy. Whereas, more than 70% of patients will experience tumor recurrence, and the majority eventually becomes treatment resistant. Ovarian cancer stem cells (CSCs) are thought to drive the universal recurrence of ovarian cancer, as well as responsible for the development of therapeutic resistance. However, the core signaling pathways regulating Ovarian CSCs remain unclear, and there is still a lack of effective drugs and drug combinations to differentiate and eliminate them to improve cancer survival.

In this study, we propose to identify potential core signaling pathways of OCSCs in a data-driven manner by integrating transcriptome data of OCSCs isolated based on two distinctive cell surface markers, ALDH and side population, with prior knowledge, e.g., regulatory network and signaling pathways, to increase the possibility of identifying the true CSC driver genes and signaling pathways. We identified 22 activated transcription factors, and 15 of them have been reported in the association with cancer stem cells. In addition, 10 transcription factors were found in the KEGG signaling pathways, and we extracted the up-stream signaling cascades regulating these transcription factors, which provide potential core signaling mechanism of ovarian CSC regulation. Moreover, we mapped the FDA approved drugs on these up-stream signaling cascades. Forty FDA approved drugs were identified and 15 of these drugs have been reported in cancer stem cell treatment. Combinations of these drugs targeting on different up-stream signaling cascades might be effective to eliminate ovarian cancer stem cells.

The proposed approach can be helpful for discovering synergistic and effective drug combinations. It is well known that inhibiting a single target does not ensure the success of effective treatment due to the complicated interplay of multiple signaling pathways [4]. For example, the activation of Sonic Hedgehog (SHH) signaling and evolution through a mesenchymal phenotype have been uncovered as a novel mechanism of drug resistance to tyrosine kinase inhibitors (TKI) of EGF receptor (EGFR) in lung cancer [48], and play important roles in regulating hepatocellular carcinoma (HCC) [58]. Also, it was reported that the number of CSCs can be increased by MSCs [59], which could be produced by the activation of SHH signaling [60] in ovarian cancer. Thus drug combinations blocking the signaling interplay have high possibility to be synergistic and effective in cancer treatment. For example, Metformin (widely used as anti-diabetic drug, also identified in this study) and MEK-inhibitors (Selumetinib/Pimasertib targeting on the RAF/RAS/MAPK signaling) were discovered to effectively inhibit the proliferation and metastasis of LKB1 positive Non-Small Cell Lung Cancer (NSCLC) cancer cells [61]. The synergism of the combination is the down-regulation of GLI1, which is the mediator of epithelia-to-mesenchymal transition (EMT) signaling, and can be affected by SHH signaling [61]. Moreover, the drug combination, Metformin and Erlotinib (EGFR inhibitor), is used in a phase II study for the treatment of stage IV NSCLC [62, 63].

There are also some limitations of this integrative data analysis. First, the current TF-target interactome data might not be complete and accurate. In the future work, we will integrate new TF-target interaction data resources, e.g., TRUST (text mining) [64] and GTRD (Chip-Seq data) [65], to improve the quality and completeness [66]. Moreover, the tissue specific regulatory network data might be useful to further refine the TF-target interactome data [66, 67]. Second, 12 activated TFs are still missed in the up-stream signaling network analysis. Additional signaling pathway database, e.g., BioGRID [68], STRING [69], Reactome [70], could be integrated to identify more up-stream signaling cascades of additional active TFs. Thirdly, other pharmacological data resources, e.g., LINCS (reverse gene signature based data) [71], can be integrated to identify more drugs or prioritize drugs to eliminate the Ovarian CSCs. Also, the drug repositioning [72–74] and drug combination prediction [75–77] are not trivial tasks. In the future work, we will integrate additional data resources to prioritize targets and drug combinations to block multiple TF and signaling interplays to eliminate ovarian CSCs.
